# Proposal of Two Prognostic Models for the Prediction of 10-Year Survival after Liver Resection for Colorectal Metastases

**DOI:** 10.1155/2018/5618581

**Published:** 2018-10-21

**Authors:** Ulf Kulik, Mareike Plohmann-Meyer, Jill Gwiasda, Joline Kolb, Daniel Meyer, Alexander Kaltenborn, Frank Lehner, Jürgen Klempnauer, Harald Schrem

**Affiliations:** ^1^General, Visceral and Transplantation Surgery, Hannover Medical School, Germany; ^2^Core Facility Quality Management & Health Technology Assessment in Transplantation, Integrated Research and Treatment Center Transplantation (IFB-Tx), Hannover Medical School, Germany

## Abstract

**Background:**

One-third of 5-year survivors after liver resection for colorectal liver metastases (CLM) develop recurrence or tumor-related death. Therefore 10-year survival appears more adequate in defining permanent cure. The aim of this study was to develop prognostic models for the prediction of 10-year survival after liver resection for colorectal liver metastases.

**Methods:**

N=965 cases of liver resection for CLM were retrospectively analyzed using univariable and multivariable regression analyses. Receiver operating curve analyses were used to assess the sensitivity and specificity of developed prognostic models and their potential clinical usefulness.

**Results:**

The 10-year survival rate was 15.2%. Age at liver resection, application of chemotherapies of the primary tumor, preoperative Quick's value, hemoglobin level, and grading of the primary colorectal tumor were independent significant predictors for 10-year patient survival. The generated formula to predict 10-year survival based on these preoperative factors displayed an area under the receiver operating curve (AUROC) of 0.716. In regard to perioperative variables, the distance of resection margins and performance of right segmental liver resection were additional independent predictors for 10-year survival. The logit link formula generated with pre- and perioperative variables showed an AUROC of 0.761.

**Conclusion:**

Both prognostic models are potentially clinically useful (AUROCs >0.700) for the prediction of 10-year survival. External validation is required prior to the introduction of these models in clinical patient counselling.

## 1. Introduction

Colorectal liver metastases (CLM) are one of the most common indications for hepatic surgery worldwide. In contrast to interventional treatment methods like radiofrequency ablation (RFA) the surgical treatment remains the only therapeutic option providing histological proven complete resection and mean 5-year survival rates of up to 50% [1–3]. Despite these encouraging results 5-year survival does not equate a permanent cure of the disease; several studies report that one-third of 5-year survivors appear to experience recurrence or tumor-related death [4–6]. Therefore, it seems more likely that 10-year survival after hepatic resection for CLM appears more qualified to be associated with permanent cure. A meta-analysis by Abbas et al. in 2011 reported 12-36% for 10-year survival rate; another study described a 10-year survival rate of 24% [7, 8]. In those reports, presence of positive resection margins clearly excluded patients from 10-year long-term survival. Furthermore extrahepatic disease and a high clinical risk score (CRS) derived from factors like carcinoembryonic antigen (CEA) levels, number and size of hepatic lesions, and the primary lymph node status were associated with reduced probability of long-term survival [7, 8]. Nevertheless, the estimation of individual prediction of long-term survival and especially a possible permanent cure is difficult and not well described in recent literature.

However, throughout the last decades several prognostic factors that influence overall survival (OS) after liver resection for CLM were reported. Size of CLM >50 mm, >1 lesion, age >70 years at liver resection, preoperative anemia, and other factors have been reported to be associated with negative impact on OS [9]. Some variables have been associated with a beneficial effect on OS, i.e., clear resection margins and the performance of only minor hepatic resections [10]. Additionally, the resection-severity-index (RSI) was recently introduced by our group as a new independent prognostic factor for survival after liver resection for CLM [11]. All those factors have usually been analyzed as regards the overall outcome after hepatic surgery for CLM while it remains unclear whether long-term survival of more than 10 years can be predicted with a prognostic model. Therefore the aim of this study was to analyze cases after hepatic surgery for CLM in a large German tertiary referral center for hepatobiliary surgery to determine patterns of pre- and perioperative factors that enable the prediction of long-term survival of ≥10 years.

## 2. Patients and Methods

### 2.1. Data Collection

This is a single center retrospective analysis. The setting of this study is a German tertiary referral center for hepatobiliary surgery and liver transplantation. The postoperative observational period ended on 27.07.2015. Descriptive statistics comparing patients with survival <10 years and survival ≥10 years are summarized in Supplementary Table 1 for preoperative variables and in Supplementary Table 2 for perioperative variables.

### 2.2. Inclusion and Exclusion Criteria

**Figure 1:**
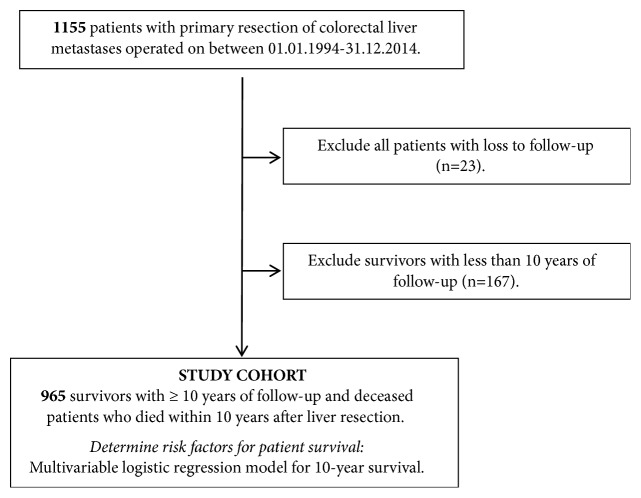
Depicted is the study flow chart of analyzed patients.

All consecutive primary liver resections for colorectal metastases performed at our institution between 01.01.1994 and 31.12.2014 (n=1155) were included. Excluded were all cases with lack of sufficient follow-up data (n=23). Furthermore all survivors with less than 10 years of follow-up (n=167) were excluded. Compliant with the STARD guidelines the analytical flow chart of the analyzed study cohort is illustrated in Figure 1 [12].

### 2.3. Ethical Considerations

The Ethics Committee of Hannover Medical School approved of this retrospective study (approval decision number 3233-2016). Patients provided informed consent that their data may be used for scientific purposes at the time of hospital admission which is the general policy of our institution. Patient records and patient data were anonymized and deidentified prior to analysis.

### 2.4. Study End-Points

The primary study end-point was observed as 10-year patient survival after liver resection (Figure 1). Patients with survival but follow-up less than 10 years cannot be included in this analysis, because we do not know whether they actually survived for 10 years or less.

### 2.5. Statistical Methods

Risk factors for patients' mortality within ten years after liver resection were analyzed with univariable and multivariable regression analyses.

Two risk-adjusted multivariable logistic regression models were developed using purposeful selection of preoperative covariates and pre-, intra-, and early postoperative covariates with p values in univariable regression ≤0.200 with the goal of avoiding overfitting and facilitating the detection of potential factor interactions based on the recommendations as published by Hosmer et al. [13]. Principal component analysis was used to identify two-sided variable correlations ≥ |0.500| to trigger a clinically informed decision on the exclusion of one of two highly correlated variables from multivariable regression in order to avoid collinearity in regression.

For all statistical tests a p value <0.05 was defined as significant. Binary variables and their influence on 10-year survival (yes/no) were analyzed with Chi^2^ tests while the influence of continuous variables on 10-year survival (yes/no) was analyzed with the Wilcoxon test.

Receiver operating characteristic curve (ROC-curve) analyses were used to assess the sensitivity and specificity of predictions derived from the final multivariable regression models and their potential usefulness as prognostic models.

The software package JMP Pro 13.0.0 (SAS Institute, Cary, NC, USA) was used to perform all statistical analyses.

## 3. Results

### 3.1. 10-Year Survival

Out of the N=965 cases finally included in the study, N=147 cases experienced long-term survival of ≥10 years (15.2%).

### 3.2. Preoperative Risk Factors for 10-Year Survival after Liver Resection

Univariable logistic regression analysis revealed that the age at operation of the primary colorectal tumor, the age at liver resection in years, the localization of the primary tumor in the colon sigmoideum (yes/no), the pT staging of the primary tumor, the pN staging of the primary tumor, the grading of the primary tumor, UICC staging of the primary tumor, chemotherapy and/or radiotherapy of the primary tumor prior to liver resection (yes/no), and the preoperative Quick's value in % all had a significant influence on 10-year survival after liver resection (Table 1).

### 3.3. Intra- and Early Postoperative Risk Factors for 10-Year Survival after Liver Resection

Univariable logistic regression analysis demonstrated that bilateral atypical liver resection, right segmental liver resection, the duration of Pringle's procedure in min, postoperative complications during hospital stay (yes/no), the size of largest metastases in mm, and the distance of the resection margin in mm to the tumor all had a significant influence on 10-year survival after liver resection (Table 2).

### 3.4. Results of Principal Component Analysis

Principal component analysis of variables with p values <0.200 in univariable logistic regression analysis demonstrated two-sided factor correlations R >|0.500| for age at operation of the primary colorectal tumor and age at liver resection in years (R=0.979), localization of the primary tumor in the colon sigmoideum (yes/no) and the rectum (yes/no) (R=0.522), UICC staging of the primary tumor and pN staging of the primary tumor (R=0.592), UICC staging of the primary tumor and the M1 stage of the primary tumor (yes/no) (R=0.761), the weight of resected liver specimen in kg, and the size of the largest metastasis in mm (R=0.584). All other variables demonstrated low two-sided factor correlations R <|0.500|.

These results lead to the decision to include the variables age at resection of liver metastases, localization of the primary tumor in the colon sigmoideum (yes/no), UICC staging of the primary tumor, and the size of the largest metastasis in mm into multivariable logistic regression analysis and to exclude the variables age at resection of primary tumor, localization of the primary tumor in the rectum, pN staging of the primary tumor, M1 stage of the primary tumor (yes/no), and the weight of resected liver specimen (kg).

### 3.5. Independent Preoperative Risk Factors for 10-Year Survival after Liver Resection

**Figure 2:**
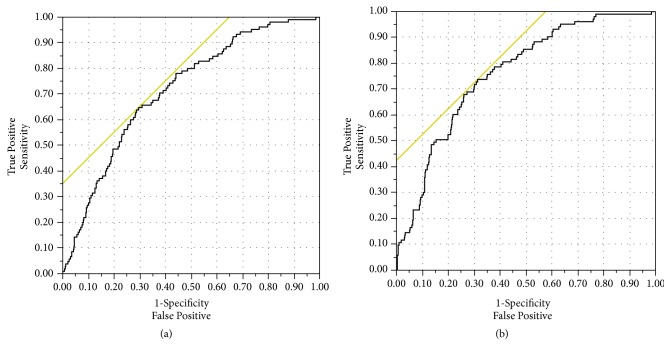
(a) Shown is the result of ROC-curve analysis of the final multivariable regression model with* preoperative* risk factors for 10-year survival (AUROC = 0.716). (b) Shown is the result of ROC-curve analysis of the final multivariable regression model with* preoperative and perioperative* risk factors for 10-year survival (AUROC = 0.761).

The finally determined logistic regression model demonstrated that age at liver resection (years), chemotherapy of the primary tumor, preoperative Quick's value in %, and hemoglobin in g/dl as well as the grading of the primary colorectal tumor were independent significant risk factors for 10-year patient survival (Table 3(a)). This model exhibited an area under the receiver operating curve (AUROC) >0.700 indicating a potential prognostic model for the prediction of 10-year survival (AUROC = 0.716) (Figure 2(a)).

This model with preoperative variables resulted in the following logit link formula:

  y = 2.893 + (0.038 *∗* age at resection of metastases in years) + (0.755 *∗* grading of the primary tumor, G1-3) + (-0.444, if no chemotherapy of the primary tumor was given—otherwise 0) + (0.444, if chemotherapy of the primary tumor was given—otherwise 0) + (-0.209 *∗* preoperative hemoglobin in g/dl) + (-0.022 *∗* preoperative Quick's value in %)

The formula for the calculation of the predicted 10-year mortality risk in % after liver resection using the logit link formula described above for preoperative variables is as follows:

  10-year mortality risk (%) = 1/(1 + Exp_(-y)_)

### 3.6. Independent Pre-, Intra-, and Early Postoperative Risk Factors for 10-Year Survival after Liver Resection

The finally determined logistic regression model demonstrated that the age at liver resection in years, the distance of the tumor to resection margin in mm, chemotherapy of the primary tumor, right segmental liver resection (yes/no), preoperative Quick's value in % and hemoglobin in g/dl, grading of the primary colorectal tumor (G1-3), and pT1-4 stage of the primary colorectal carcinoma were independent significant risk factors for 10-year patient survival (Table 3(b)). This model exhibited an area under the receiver operating curve (AUROC) >0.700 indicating a potential prognostic model for the prediction of 10-year survival (AUROC = 0.761) (Figure 2(b)).

This model with pre-, intra-, and early postoperative variables resulted in the following logit link formula:

  y = 1.391 + (0.043 *∗* age at resection of metastases in years) + (0.3502 *∗* pT stage of the primary tumor, pT1-4) + (0.723 *∗* grading of the primary tumor, G1-3) + (-0.387, if no chemotherapy of the primary tumor was given—otherwise 0) + (0.387, if chemotherapy of the primary tumor was given—otherwise 0) + (-0.204 *∗* preoperative hemoglobin in g/dl) + (-0.021 *∗* preoperative Quick's value in %) + (0.575, if no right segmental liver resection was performed—otherwise 0) + (-0.575, if right segmental liver resection was performed—otherwise 0) + (-0.033 *∗* distance of liver metastasis to resection margin in mm)

Calculation of the predicted 10-year mortality risk in % after liver resection using the logit link formula described above for pre-, intra-, and early postoperative variables is as follows:

  10-year mortality risk (%) = 1/(1 + Exp_(-y)_)

## 4. Discussion

This study identified factors with an independent significant influence on long-term survival of ≥10 years after hepatic surgery for CLM in a large collective including 147 patients who survived at least 10 years. Two prognostic models for the prediction of the probability of experiencing that long-term survival are proposed. The proposed models are specific to estimate 10-year survival after liver resection. The first model is based on preoperative factors and offers the chance to estimate possible 10-year survival before performance of the liver surgery, for example, when meeting a patient in the outpatient clinic (Figure 2(a)). The second model includes factors from the surgery and the early postoperative course and opens a more detailed view based on the specific liver surgery that was performed and the results of the histopathology (Figure 2(b)). That model might enable a more elaborated design of the medical aftercare. Up to now, no prognostic model was available to estimate the odds for long-term survival of ≥10 years after liver resection for CLM. Most recently published studies only aimed for the assessment of long-term survival rates and risk factors that generally influence long-term or overall survival. The 10-years survival rate of 15.2% found in our study matches the range of long-term survival reported in that current literature [7, 8].

Based on the first preoperative model the odds for 10-year survival are better when the patients are younger, showed a low primary tumor grading, did not receive a chemotherapy of the primary tumor, and displayed higher Hb-values and Quick values preoperatively (Table 3(a)). We believe that an as accurate as possible prediction of the likelihood of 10-year survival before liver surgery may play a role for many patients to understand the chances of cure after surgery. The influence of the patients' age on overall survival is well described; various studies reported limited overall survival in elderly patients. Nevertheless outcome is still far better than without surgical treatment of CLM [9, 14, 15]. Similar findings were repeatedly published as regards the primary tumor grading; a G3-grading is usually associated with decreased survival [16, 17]. Likewise, a lower Hb-value was recently reported by our center as negative predictor for survival [18]. Furthermore, a lower Hb-value might cause an increased need for perioperative blood transfusion, lately reported to impair disease-free and overall survival [19, 20]. The Quick's value in % was not described previously as prognostic factor in liver resection for colorectal liver metastases but it appears logical that a higher value correlates with a better synthetic function of the organ and a more stable function of the liver remnant after resection.

The negative influence of chemotherapies applied in the context of the primary tumor appears more surprising. It can be speculated that patients who received chemotherapies showed an initially higher UICC-stage and displayed synchronous, possibly nonresectable liver metastases. Hence, the chemotherapy might be considered as a surrogate parameter for a more advanced disease with corresponding impaired outcome. In contrast to this notion, the correlation of chemotherapy for the primary tumor with more advanced disease stages of the primary tumor was low in this study (R <|0.500|). Unfortunately, the heterogeneity of chemotherapy protocols given over the study period and the different approaches (neoadjuvant, adjuvant, or initially palliative as regards synchronous nonresectable CLM) limits the statistically convincing analysis; therefore a deeper analysis in our cohort was disregarded. Of note, chemotherapy of primary tumor in this study defines chemotherapy with any purpose that was delivered before liver resection. This may have created to some degree a bias in the results of this investigation.

Nevertheless, another group reported an inferior outcome after resection for CLM in these cases with chemotherapeutical treatment prior to liver resection. In this study a chemotherapy was significantly more often applied when synchronous liver metastases or more than three liver lesions were diagnosed [21]. Furthermore, over the last years chemotherapies are more frequently used as regards downsizing liver lesions and to converse initially nonresectable into resectable CLM (conversion chemotherapy). For instance, following the use of fluorouracil, leucovorin, oxaliplatin, and irinotecan regimes (FOLFOXIRI) a secondary resectability of CLM of 36% was described [22]. Reasonable outcome was reported with 2-year survival of 83% and 5-year survival rate of 33-50% after a conversion therapy and secondary liver resection [23–25]. In all these studies the majority of patients displayed synchronous liver metastases; therefore those chemotherapies might be considered as adjuvant or with initial palliative intent as regards the primary tumor. Furthermore, the negative impact of chemotherapies on long-term survival might be related to chemotherapy associated liver damage (CALI). Various agents drive several mechanisms of hepatic injury; e.g., oxaliplatin is associated with the sinusoidal obstruction syndrome (SOS), 5-fluorouracil is known to cause steatosis, and irinotecan may induce steatohepatitis [26]. Those toxicities might be linked to increased postoperative morbidity after liver resection [27, 28]. Taken together, the association of chemotherapy with inferior odds for 10-year survival is most likely related to advanced primary disease and may be a possible consequence of chemotherapy induced liver damage. However, it can be assumed that such cases with advanced diseases would be most likely associated with an even worse prognosis without chemotherapy of the primary tumor.

The second model is expanded by parameters of the surgical resection and the histopathology. Basically in addition to the preoperative model the odds for 10-year survival are better in case of a right segmental resection and wider distance of liver metastasis to the resection margin (Table 3(b)). As preluded, positive resection margins were previously identified as risk factor with negative influence on long-term survival [7]. The same effect was repeatedly reported regarding the overall survival: A positive resection margin or a distance of <1 mm to the metastasis was associated with inferior 5-year survival or overall outcome [29–31]. In our cohort no definite benefit of a R0 resection in comparison to R1 resection was observed in the univariable analyses but in the multivariable model wider distance of metastasis to the resection margins is clearly associated with better outcome. Further research to define possible cut-off values of margin width that are associated with poorer outcome or no more benefit on survival is needed. Nevertheless, the strong effect on long-term survival is presumably related to higher rates of tumor recurrence limiting subsequent treatment options.

As regards the beneficial effect of right segmental resections the interpretation is more difficult. It has been previously described that minor liver resections are associated with a better outcome, possibly because of a larger liver remnant with more stable liver function and the technical possibility of future resections in case of recurrence [10, 32]. On the other hand, what favors a right segmental resection in contrast to other minor resections, like nonanatomic right/left or a left segmental resection, is unclear. In that context, one older study showed no influence on overall survival in comparison of nonanatomical and anatomical minor liver resections [33]. Nevertheless, the right liver appears to be more commonly affected by CLM than the left liver, probably due to the more right oriented portal vein flow [34]. Speculatively, in case of a right segmental resection the chances that the metastasis there is truly the only lesion and no other occult nodes are present in the left lobe and that possible subdetectable lesions in the same segment are also removed might be better than in case of a nonanatomical resection with subsequent higher odds for long-term survival of >10 years.

In summary, this work proposes prognostic models for the prediction of the likelihood of long-term survival of >10 years after liver resection for CLM based on easy to access pre- and perioperative factors. Of course the retrospective approach and the single-center nature of this study limit the generalizability of the findings. Therefore the results need to be reevaluated by others to exclude a center-bias. The proposed prognostic models warrant external model validation.

## Figures and Tables

**Table 1:** Shown are the results of univariable binary logistic regression to determine the influence of each *preoperative* variable on 10-year survival after liver resection. Odds ratios greater than 1 with a significant p value (p<0.05) indicate variables that increase the risk of mortality within 10 years significantly whereas odds ratios smaller than 1 with a significant p value indicate variables that decrease the risk of mortality within 10 years.

**Variables**			**Odds Ratio**	**95**%**-Confidence interval**	**p-value**
**Pre-operative variables**	Female gender (yes/no)		0.986	0.687 – 1.416	0.941
	Male gender (yes/no)		1.014	0.706 – 1.455	0.941
	Age at operation of primary tumor (years)		1.029	1.013 – 1.046	**<0.001**
	Age at liver resection (years)		1.028	1.012 – 1.045	**<0.001**
	Time between resection of primary tumor and resection of metastases (days)		1.000	0.999 – 1.000	0.900
	Localization of primary tumor	Coecum (yes/no)	2.119	0.902 – 4.980	**0.058**
		Colon ascendens (Yes/no)	0.895	0.528 – 1.517	0.683
		Colon transversum (yes/no)	1.061	0.438 – 2.570	0.895
		Colon descendens (yes/no)	1.136	0.568 – 2.269	0.715
		Colon sigmoideum (yes/no)	0.659	0.462 – 0.941	**0.023**
		Rectum (yes/no)	1.314	0.891 – 1.939	**0.162**
	Stage and grading of primary tumor	pT1-4 (ordinal scale)	not determined		**0.004**
		pT1 vs. pT2	0.965	0.375 – 2.478	0.940
		pT1 vs. pT3	0.610	0.255 – 1.454	0.264
		pT1 vs. pT4	0.214	0.069 – 0.670	**0.008**
		pN0-2b (ordinal scale)	not determined		**0.034**
		pN0 vs. pN1	0.587	0.380 – 0.906	**0.016**
		pN0 vs. pN2a	0.551	0.335 – 0.907	**0.019**
		pN0 vs. pN2b	0.912	0.385 – 2.158	0.834
		M1 (yes/no)	1.489	1.017 – 2.181	**0.038**
		Grading G1-3 (ordinal scale)	not determined		**0.012**
		Grading G1 vs. G2	0.324	0.140 – 0.750	**0.009**
		Grading G1 vs. G3	0.162	0.054 – 0.481	**0.001**
		UICC I-IV (ordinal scale)	not determined		**0.016**
		UICC I vs. IIa	0.438	0.227 – 0.844	**0.014**
		UICC I vs. IIb	0.206	0.025 – 1.687	**0.141**
		UICC I vs. IIIa	0.452	0.166 – 1.235	**0.122**
		UICC I vs. IIIb	0.312	0.154 – 0.633	**0.001**
		UICC I vs. IIIc	0.422	0.207 – 0.862	**0.018**
		UICC I vs. IV	0.318	0.178 – 0.567	**<0.001**
	Chemotherapy of primary tumor (yes/no)		1.831	1.281 – 2.618	**<0.001**
	Radiotherapy of primary tumor (yes/no)		1.847	0.991 – 3.444	**0.039**
	Local recurrence of primary tumor (yes/no)		1.229	0.617 – 2.447	0.549
	Simultaneous resection of primary tumor and liver metastases (yes/no)		0.979	0.502 – 1.909	0.950
	Multiple resection of metastases (yes/no)		1.131	0.624 – 2.048	0.682
	Leukocytes Tsd/*μ*l		1.035	0.954 – 1.124	0.397
	Platelets Tsd/*μ*l		0.999	0.997 – 1.000	**0.155**
	Hemoglobin g/dl		0.891	0.789 – 1.007	**0.062**
	Quick's value %		0.979	0.966 - 0.992	**0.002**

**Table 2:** Shown are the results of univariable binary logistic regression to determine the influence of each *intraoperative* variable on 10-year survival after liver resection. Odds ratios greater than 1 with a significant p value (p<0.05) indicate variables that increase the risk of mortality within 10 years significantly whereas odds ratios smaller than 1 with a significant p value indicate variables that decrease the risk of mortality within 10 years.

**Variables**		**Odds Ratio**		**95**%**-Confidence interval**	**p-value**
**Intra-operative variables**	Left atypical liver resection	1 point	1.155	0.537 – 2.488	0.708
	Right atypical liver resection		0.947	0.621 – 1.444	0.801
	Bilateral atypical liver resection	2 points	2.448	1.165 – 5.142	**0.008**
	Left segmental liver resection		0.764	0.397 – 1.468	0.430
	Right segmental liver resection		0.352	0.192 – 0.645	**0.002**
	Left hemihepatectomy	3 points	2.477	0.883 – 6.951	0.512
	Right hemihepatectomy	4 points	0.781	0.521 – 1.171	0.238
	Extended left hepatectomy	5 points	1.114	0.645 – 1.922	0.696
	Left hepatectomy and right atypical liver resection	6 points	-	-	-
	Extended right hepatectomy		1.145	0.475 – 2.759	0.760
	Right hepatectomy and left atypical liver resection	7 points	1.034	0.352 – 3.035	0.951
	Extent of resection (ordinal scale)	not determined			0.455
	Extent of resection 1 point vs. 2 points	1.084		0.668 – 1.760	0.745
	Extent of resection 1 point vs. 3 points	0.418		0.143 – 1.217	**0.110**
	Extent of resection 1 point vs. 4 points	1.202		0.741 – 1.949	0.455
	Extent of resection 1 point vs. 5 points	0.905		0.491 – 1.668	0.749
	Extent of resection 1 point vs. 6 points	0.707		0.284 – 1.760	0.456
	Extent of resection 1 point vs. 7 points	0.963		0.316 – 2.930	0.947
	Operative duration in min	1.001		0.999 – 1.002	0.568
	Duration of Pringle's procedure in min	0.983		0.973 – 0.994	**0.003**
	Complications yes/no	1.734		1.028 – 2.925	**0.030**
	Intraoperative transfusion of units of packed red blood cells	1.078		1.001 – 1.161	**0.031**
	Size of largest metastasis in mm	1.005		1.000 – 1.011	**0.039**
	Weight of liver specimen in kg	1.297		0.868 – 1.939	**0.188**
	Distance to resection margin in mm	0.972		0.960 – 0.986	**<0.001**
	Grading G1-G3 (ordinal scale)	not determined			**0.173**
	Grading G1 vs. G2	1.873		0.238 – 14.749	0.551
	Grading G1 vs. G3	0.732		0.069 – 7.799	0.796
	R-status R0 (yes/no)	0.363		0.086 – 1.537	**0.111**

**Table:** (a) Shown is the final multivariable model with *preoperative* risk factors only for 10-year survival after liver resection. Odds ratios greater than 1 with a significant p value (p<0.05) indicate variables that increase the risk of mortality within 10 years independently and significantly whereas odds ratios smaller than 1 with a significant p value indicate variables that decrease the risk of mortality within 10 years independently and significantly

**Variables**	**Odds Ratio**	**95**%**-Confidence interval**	**p-value**
Chemotherapy of primary tumor (yes/no)	2.432	1.570 – 3.770	**<0.001**
Age at liver resection (years)	1.039	1.019 – 1.060	**<0.001**
Quick's value %	0.979	0.964 – 0.993	**0.005**
Hemoglobin g/dl	0.811	0.698 – 0.943	**0.006**
Grading of primary tumor G1-3 (ordinal scale)	2.127	1.108 – 4.081	**0.019**

**Table:** (b) Shown is the final multivariable model with *preoperative and perioperative* risk factors for 10-year survival after liver resection. Odds ratios greater than 1 with a significant p value (p<0.05) indicate variables that increase the risk of mortality within 10 years independently and significantly whereas odds ratios smaller than 1 with a significant p value indicate variables that decrease the risk of mortality within 10 years independently and significantly

**Variables**	**Odds Ratio**	**95**%**-Confidence interval**	**p-value**
Age at liver resection (years)	1.044	1.022 – 1.066	**<0.001**
Distance to resection margin in mm	0.968	0.950 – 0.986	**<0.001**
Chemotherapy of primary tumor (yes/no)	2.168	1.368 – 3.435	**<0.001**
Right segmental liver resection	0.317	0.152 – 0.661	**0.003**
Quick's value %	0.980	0.964 – 0.994	**0.006**
Hemoglobin g/dl	0.815	0.700 – 0.955	**0.010**
Grading of primary tumor G1-3 (ordinal scale)	2.060	1.050 – 4.037	**0.031**
pT1-4 primary tumor (ordinal scale)	1.420	1.013 – 1.990	**0.044**

## Data Availability

The clinical data used to support the findings of this study are included within the article and the supplementary information.

## Supplementary Materials

Supplementary Materials Supplementary information regarding the distribution of* preoperative* data between patients who died within 10 years after resection and those who survived more than 10 years is provided in Supplementary Table 1. Supplementary Table 2 displays the distribution of* intraoperative* variables between patients who died within 10 years after resection and those who survived more than 10 years. In both tables data is summarized showing the frequencies n (%) for positive binary data and the median (mean, min. - max.) for continuous data.
