# Supplementary material for: Proposal of Two Prognostic Models for the Prediction of 10-Year Survival after Liver Resection for Colorectal Metastases
Source: HPB Surg. 2018 Oct 21;2018:5618581. doi: 10.1155/2018/5618581 (PMC6215566; doi:10.1155/2018/5618581)
Supplement: Supplementary Materials — Supplementary information regarding the distribution of preoperative data between patients who died within 10 years after resection and those who survived more than 10 years is provided in Supplementary Table 1. Supplementary Table 2 displays the distribution of intraoperative variables between patients who died within 10 years after resection and those who survived more than 10 years. In both tables data is summarized showing the frequencies n (%) for positive binary data and the median (mean, min. - max.) for continuous data. [file 5618581.f1.docx]

| **Variables** | | **Survival ≤ 10 years after resection of**  **liver metastases**  **n=818** | **Survival > 10 years after resection of**  **liver metastases**  **n=147** | **p-value**  * Chi²-test  ** Wilcoxon-test | **Missing**  **values** |
| --- | --- | --- | --- | --- | --- |
| **Pre-operative variables** | Female gender | 309 (37.8%) | 56 (38.1%) | 0.941* | 0 (0%) |
|  | Male gender | 509 (62.2%) | 91 (61.9%) | 0.941* | 0 (0%) |
|  | Age at operation of primary tumor (years) | 61.0 (60.1; 24.4 – 88.0) | 57.2 (56.7; 21.9 – 77.0) | **<0,001**** | 6 (0.6%) |
|  | Age at liver resection (years) | 63.4 (61.9; 26.8 – 90.7) | 59.4 (58.6; 23.9 – 79.0) | **<0.001**** | 0 (0%) |
|  | Time between resection of primary tumor and resection of metastases (days) | 437 (666; 0 -8494) | 568 (675.7; 0 – 4530) | 0.205** | 6 (0.6%) |
|  | Coecum | 67 (8.3%) | 6 (4.1%) | **0.078*** | 8 (0.8%) |
|  | Colon ascendens | 95 (11.7%) | 19 (12.9%) | 0.680* | 8 (0.8%) |
|  | Colon transversum | 35 (4.3%) | 6 (4.1 %) | 0.895* | 8 (0.8%) |
|  | Colon descendens | 62 (7.7%) | 10 (6.8%) | 0.719* | 8 (0.8%) |
|  | Colon sigmoideum | 278 (34.3%) | 65 (44.2%) | **0.021*** | 8 (0.8%) |
|  | Rectum | 273 (33.7%) | 41 (27.9%) | **0.167*** | 8 (0.8%) |
|  | pT1 | 24 (3.2%) | 7 (5.2%) | 0.233* | 68 (7.0%) |
|  | pT2 | 96 (12.6%) | 27 (20.0%) | **0.021*** | 68 (7.0%) |
|  | pT3 | 529 (69.4%) | 94 (69.6%) | 0.962* | 68 (7.0%) |
|  | pT4 | 112 (14.7%) | 7 (5.2%) | **0.003*** | 68 (7.0%) |
|  | pN0 | 271 (35.6%) | 65 (48.1%) | **0.005*** | 68 (7.0%) |
|  | pN1 | 270 (35.4%) | 38 (28.1%) | **0.100*** | 68 (7.0%) |
|  | pN2a | 189 (24.8%) | 25 (18.5%) | **0.114*** | 68 (7.0%) |
|  | pN2b | 32 (4.2%) | 7 (5.2%) | 0.605* | 68 (7.0%) |
|  | M1 | 334 (44.3%) | 47 (34.8%) | **0.040*** | 58 (6.0%) |
|  | Grading G1 | 16 (2.1%) | 9 (6.7%) | **0.003*** | 77 (8.0%) |
|  | Grading G2 | 648 (86.1%) | 118 (87.4%) | 0.674* | 77 (8.0%) |
|  | Grading G3 | 88 (11.7%) | 8 (5.9%) | **0.047*** | 77 (8.0%) |
|  | R-status R0 | 748 (98.8%) | 135 (100.0%) | 0.203* | 73 (7.6%) |
|  | UICC I | 52 (6.8%) | 23 (17.0%) | **<0.001*** | 65 (6.7%) |
|  | UICC IIa | 124 (16.2%) | 24 (17.8%) | 0.650* | 65 (6.7%) |
|  | UICC IIb | 11 (1.4) | 1 (0.7%) | 0.515* | 65 (6.7%) |
|  | UICC IIIa | 30 (3.9%) | 6 (4.4%) | 0.775* | 65 (6.7%) |
|  | UICC IIIb | 123 (16.1%) | 17 (12.6%) | 0.303* | 65 (6.7%) |
|  | UICC IIIc | 91 (11.9%) | 17 (12.6%) | 0.818* | 65 (6.7%) |
|  | UICC IV | 334 (43.7%) | 47 (34.8%) | **0.055*** | 65 (6.7%) |
|  | Chemotherapy of primary tumor | 465 (57.8%) | 62 (42.8%) | **<0.001*** | 15 (1.6%) |
|  | Radiotherapy of primary tumor | 115 (14.3%) | 12 (8.3%) | **0.050*** | 15 (1.6%) |
|  | Local recurrence of primary tumor | 67 (8.2%) | 10 (6.8%) | 0.557* | 4 (0.4%) |
|  | Simultaneous resection of primary tumor and liver metastases | 60 (7.3%) | 11 (7.5%) | 0.950* | 0 (0%) |
|  | Multiple resection of metastases | 87 (10.6%) | 14 (9.5%) | 0.685* | 0 (0%) |
|  | Leukocytes Tsd/µl | 6.8 (7.1; 1.9 – 29.2) | 6.6 (7.0; 3 – 13.6) | 0.563** | 45 (4.7%) |
|  | Platelets Tsd/µl | 242 (254.9; 8 – 977) | 263 (266.9; 107 – 542) | **0.005**** | 45 (4.7%) |
|  | Hemoglobin g/dl | 13.5 (13.4; 7.9 – 18.3) | 13.8 (13.7; 9.3 – 17) | **0.049**** | 45 (4.7%) |
|  | Quick % | 100 (100; 33 – 159) | 104 (105.0; 42 – 165) | **0.004**** | 189 (19.6%) |

**Supplementary Table 1:** Shown is the distribution of *pre-operative* data between patients who died within 10 years after resection versus those who survived more than 10 years. Data is summarized showing the frequencies n (%) for positive binary data and the median (mean, min. - max.) for continuous data.

| **Variables** | | | **Survival ≤ 10 years after resection of liver metastases**  **n=818** | **Survival > 10 years after resection of liver metastases**  **n=147** | **p-value**  * Chi²-test  ** Wilcoxon-test | **Missing**  **values**  **n (%)** |
| --- | --- | --- | --- | --- | --- | --- |
| **Extent of resection** | Left atypical liver resection | 1 point | 51 (6.2%) | 8 (5.4%) | 0.712* | 0 (0%) |
|  | Right atypical liver resection |  | 176 (21.5%) | 33 (22.4%) | 0.800* | 0 (0%) |
|  | Bilateral atypical liver resection | 2 points | 101 (12.3%) | 8 (5.4%) | **0.015*** | 0 (0%) |
|  | Left segmental liver resection |  | 52 (6.4%) | 12 (8.2%) | 0.418* | 0 (0%) |
|  | Right segmental liver resection |  | 36 (4.4%) | 17 (11.6%) | **<0.001*** | 0 (0%) |
|  | Left hemihepatectomy | 3 points | 53 (6.5%) | 4 (2.7%) | **0.075*** | 0 (0%) |
|  | Right hemihepatectomy | 4 points | 175 (21.4%) | 38 (25.9%) | 0.230* | 0 (0%) |
|  | Extended left hepatectomy | 5 points | 104 (12.7%) | 17 (11.6%) | 0.699* | 0 (0%) |
|  | Left hepatectomy and right atypical liver resection | 6 points | 9 (1.1%) | 0 (0%) | 0.201* | 0 (0%) |
|  | Extended right hepatectomy |  | 38 (4.6%) | 6 (4.1%) | 0.763* | 0 (0%) |
|  | Right hepatectomy and left atypical liver resection | 7 points | 23 (2.8%) | 4 (2.7%) | 0.951* | 0 (0%) |
|  | Extent of resection in points | | 2 (3; 1-7) | 2 (2.9; 1 – 7) | 0.750** | 0 (0%) |
| **Operative details** | Operative duration in min | | 190 (210.5; 57-720) | 190 (205.7; 40 – 645) | 0.982** | 10 (1.0%) |
|  | Duration of Pringle’s procedure in min | | 20 (19.9; 0-83) | 25 (24.1; 0 – 72) | **0.001**** | 11 (1.1%) |
|  | Complications yes/no | | 158 (19.5%) | 18 (12.2%) | **0.037*** | 7 (0.7%) |
|  | Intraoperative transfusion of units of packed red blood cells | | 1 (2.1; 0-40) | 0 (1.6; 0 – 9) | **0.130**** | 9 (0.9%) |
|  | Clavien Dindo Classification of surgical complications grade 1 | | 25 (3.1%) | 1 (0.7%) | **0.099*** | 7 (0.7%) |
|  | Clavien Dindo Classification of surgical complications grade 2 | | 29 (3.6%) | 2 (1.4%) | **0.163*** | 7 (0.7%) |
|  | Clavien Dindo Classification of surgical complications grade 3 | | 67 (8.3%) | 15 (10.2%) | 0.439* | 7 (0.7%) |
|  | Clavien Dindo Classification of surgical complications grade 4 | | 18 (2.2%) | 0 (0%) | **0.068*** | 7 (0.7%) |
|  | Clavien Dindo Classification of surgical complications grade 5 | | 19 (2.3%) | 0 (0%) | **0.061*** | 7 (0.7%) |
| **Characteristics and Grading of liver metastases** | Size of largest metastasis in mm | | 50 (58.6; 3-217) | 44 (51.6; 6 – 160) | **0.064**** | 61 (6.3%) |
|  | Weight of liver specimen in kg | | 0.4 (0,5; 0-4.9) | 0.4 (0.5; 0 – 69) | 0.846** | 43 (4.5%) |
|  | Distance to resection margin in mm | | 2 (6.8; 0-100) | 7 (10.9; 0 – 69) | **<0.001**** | 18 (1.9%) |
|  | Grading G1 | | 10 (1.2%) | 1 (0.7%) | 0.566* | 10 (1.0%) |
|  | Grading G2 | | 758 (93.7%) | 142 (97.3%) | **0.089*** | 10 (1.0%) |
|  | Grading G3 | | 41 (5.1%) | 3 (2.1%) | **0.110*** | 10 (1.0%) |
|  | R-status R0 | | 779 (96.3%) | 143 (98.6%) | **0.152*** | 11 (1.1%) |
|  | R-status R1 | | 27 (3.3%) | 2 (1.4%) | 0.206* | 11 (1.1%) |
|  | R-status R2 | | 3 (0.4%) | 0 (0%) | 0.463* | 11 (1.1%) |

**Supplementary Table 2:** Shown is the distribution of *intra-operative* variables between patients who died within 10 years after resection versus those who survived more than 10 years. Data is summarized showing the frequencies n (%) for positive binary data and the median (mean, min. - max.) for continuous data.
